# Is religiousness *really* helpful to reduce depressive symptoms at old age? A longitudinal study

**DOI:** 10.1371/journal.pone.0218557

**Published:** 2019-07-03

**Authors:** Lore Van Herreweghe, Wim Van Lancker

**Affiliations:** 1 Centre for Sociological Research, University of Leuven, Leuven, Belgium; 2 Herman Deleeck Centre for Social Policy, University of Antwerp, Antwerp, Belgium; Indiana University Purdue University at Indianapolis, UNITED STATES

## Abstract

**Background:**

Higher levels of religiousness are associated with better mental health outcomes, but most of previous research is cross-sectional, failing to address issues of selection and reverse causation.

**Methods:**

We assessed the longitudinal association between both public and private religiousness and depressive symptoms, drawing on data from 7,719 persons aged 65 and older of the Survey of Health, Ageing and Retirement in Europe (SHARE). Repeated measurements of different aspects of religiousness and depressive symptoms were used in random and fixed-effects models in order to assess the effect of changes in religious behavior on changes in depressive symptoms.

**Results:**

Praying more than once a day was associated with more depressive symptoms (β = 0.150, 95% CI: 0.003, 0.298) relative to individuals who never pray, adjusted for socio-demographic characteristics, physical health covariates and history of depression, but the comparison with the fixed effects model suggests that this is the result of a selection effect. Participating weekly or more in a religious organization was associated with fewer depressive symptoms (β = -0.219, 95% CI: -0.344, -0.094), but this appeared to be spurious after taking due account of possible confounders (β = -0.092, 95% CI: -0.223, 0.038). Focusing on within-persons changes, we found that participating in religious organizations weekly or more was associated with more depressive symptoms (β = 0.275, 95% CI: 0.075, 0.475).

**Conclusion:**

Our findings do not support that religious behavior, both public or private, may be beneficial for the mental health of older Europeans.

## Introduction

Depression is among the most prevalent mental disorders in middle and high income countries and it is ranked as the leading cause of disability worldwide [1]. With a rapidly ageing population, depression amongst the elderly is therefore an important global public health concern. While old age can be a time of well-deserved rest with more spare time to spend with family, to engage in social and cultural activities and to enjoy life after all those working years, unfortunately it is often accompanied with a variety of challenges which are associated with depression: loneliness, poor social support, disability, deteriorating health, financial strains and poverty [2–4]. Depression is associated with decreased well-being and negative health outcomes, such as suicide [5] and cardiac disease [6]. For that reason, understanding the social origins of depressive symptoms is of utmost importance for public policies to be effective in improving quality of life in old age.

One of such social determinants is religious participation. The majority of previous research suggests fairly consistent yet modest protective effects of religious participation on depression, especially for religious service attendance [7–11]. This inverse association can be explained through two different pathways. First, religion as a personal practice, such as praying, might be a way to cope with stress, in turn reducing the likelihood that stress will develop into depressive symptoms. At some point in later life, stressful life-events are likely to happen, like sickness, the loss of a loved one, or changes in one’s financial situation due to retirement. Given the higher frequency of occurrence of such life events in old age, religion as a personal practice may be of particular importance to the elderly [12]. Second, religion as a public activity provides social support through the communities of religious groups [13]. The support offered by these social groups can be understood as individuals who share the same interests, values and norms [14], or who assist each other with tasks, or financial assistance [12]. As social support is found to be an important condition of mental health and well-being, particularly for the older and retired population [15–17], participating in religious activities or communities might affect the risk of depression and depressive symptoms in a beneficial way. As such, religiousness as both a public social support and a personal coping strategy is an important element in the study of the social origins of mental ill-health.

Yet, studies that apply such multidimensional construct of religiosity find that particular forms of religiosity are associated with higher levels or incidence of depressive symptoms at old age [18–23]. It is mostly communal forms of religious participation rather than merely private practices, which are found to be protective against mental ill-health [8,9]. Personal aspects of religion are often portrayed as sources of solace and comfort. However, one could also experience feelings of disappointment and abandonment when they are faced with stressful or traumatic circumstances. Under such conditions, people could experience their relationship with God in an unsatisfying and punitive way [24]. Additionally, research has suggested that there are two epidemiological forces in the relationship between religiousness and mental health. People can be religious by relying intrinsically on their faith, while for others religiousness can be prompted by a certain life event and used as a coping strategy. The first can be referred to as “*restful*” religiousness, while the latter can be described as “*crisis*” religiousness [25]. The recent study of Ahrenfeldt et al. [8] found that restful religiousness (identified by praying, taking part in a religious organization and being religiously educated) was associated with good health, and that crisis religiousness (identified by praying only, without reporting religiousness in other ways) was associated with poorer than average health outcomes, suggesting that disease crisis may led people to pray. Being religiously educated could strengthen certain religious beliefs and convictions, like believing in God and life after death [25], and can be seen as an indicator of “restful” religiousness. Therefore, it’s essential to distinguish between different aspects of religiosity, since different dimensions can affect depressive symptoms at old age in different and possibly contradictory ways.

The relationship between religiousness and mental health has been a topic of interest for a long time, however the current research literature is still subject to a number of limitations. First limitation in the current body of research on religiousness and depression is that the majority of studies are cross-sectional [9] and thus based on measures at only one point in time or without control for baseline depression. However, prior depression can affect subsequent religious behavior, which means that an inverse relationship between religious service attendance and depression can be the result of depressed individuals stopping religious service attendance, and not because service attendance protects against depression [26,27]. Evidence for such reverse causality is provided by Maselko et al. [26]. They find that women diagnosed with depression before age 18 were more likely to stop attending religious services in early adulthood, compared to women with adult-onset depression or no lifetime depression. Li et al. [28] find that women suffering from depression were less likely to subsequently attend religious services once or more per week four years later, compared to women who were not depressed in the past. Due to the possibility of feedback, we see controlling for previous depression as a necessity when studying religiousness and mental health, which cannot be done based on data collected at a single point in time.

A second limitation is that the vast majority of studies were carried out in the United States (US) [9]. This is also the case for the literature on religiousness and health in general [29]. There is a small proportion of studies using longitudinal data to assess associations between religiousness and depression find protective effects of service attendance on depression in the US [28, 30–32], but much less is known about the association outside the US [33].

The US is typically found to be a moderately religious nation relative to Europe which tends to be more secular, especially Northern Europe [34]. Adding to that, research suggests that a protective association between religiousness and (mental) health can be more substantial in regions that are more religious [35, 36] and that religious people report higher levels of well-being [30, 35]. One might thus expect that the association between depressive symptoms and religious participation will be weakest or less protective in Northern Europe [33], and that the role of religion in coping with illnesses will be lower compared to the US [37]. This hypothesis is supported by the idea that the positive effects of religiosity are based on both religious group behavior and the existence of an important religious community. This means that the positive effects of religiousness rely on whether or not religion is practiced in group and on the social significance of the religious community [38]. In Europe, we see that the institutional forms, in particular the traditional Christian Churches, are increasingly losing social significance [39], while the U.S. remains predominantly Christian with three-quarters of adults identifying themselves as Christians [40]. Due to their different religious contexts, the effect of religiousness on depressive symptoms is likely to vary across Europe and the U.S. Therefore, we will add to the existing literature on religiousness and mental health by focusing on 11 European countries.

This article expands the current literature on depression and religiousness by assessing the longitudinal association between different aspects of religiousness and depressive symptoms among elderly in the European context, while accounting for potential feedback. In doing so, we expand the dominant cross-sectional between-persons approach in the current research on religiousness and depression with a longitudinal within-persons approach, as both religiousness and depressive symptoms are not fixed states, but characteristics changing over time, possibly in a causal way.

## Methods

### Sample

We draw data from the Survey of Health, Ageing and Retirement in Europe (SHARE) [41]. SHARE is a representative dataset of individuals aged 50 and older in 19 European countries and Israel. SHARE includes microdata on the socio-economic status and health status, both physical and mental, and allows the cross-national comparison of health indicators as well as the analysis of the determinants of health over time [4]. We select respondents aged 65 and older who entered the survey in wave 1 (2004–2005) and were followed-up in at least one subsequent wave (wave 2 (2006–2007), 4 (2011) or 5 (2013)). Wave 3 does not assess depressive symptoms or religiousness and is therefore excluded from the analyses. We include respondents with no missing values of depressive symptoms and religiousness at wave 1 (n = 7,719). This sample includes participants come from Austria, Germany, Sweden, Netherlands, Spain, Italy, Denmark, Greece, Switzerland, Belgium and Israel. These countries all participated in the first wave of the SHARE, and in at least one subsequent wave. France, however, was dropped as their questionnaire did not include all three measures of religiousness.

### Measures

#### Assessment of depressive symptoms

Depressive symptoms are repeatedly measured by means of the EURO-D scale in all four waves. This measurement of depression was developed by Prince et al. [42] and is a widely used validated indicator of late-life depression [43]. The score range is 0–12, with higher scores indicating higher levels of depressive symptoms. A threshold of 4 or above is used to indicate a clinically significant depression.

#### Religiousness

Religiosity is multidimensional and different aspects of religion may be associated with depression in a different way. Therefore, three measures of religiousness will be included in our models. The first two are repeatedly measured across all four waves, which allows us to assess the effect of changes in religious behavior on depressive symptoms. First, the public aspect of religiousness is measured by questions on whether the respondent has participated in a religious organization, and how often. The first question is “Have you done any of these activities in the past month?: Taken part in a religious organization (church, synagogue, mosque, etc.), which respondents could answer with “Yes” or “No”. If they did taken part in a religious organizations, they got asked the second question: “How often in the past four weeks?: Taken part in a religious organization (church, synagogue, mosque, etc.)”. Respondents could then choose between “Almost daily”, “Almost every week”, or “Less often [than weekly]”. In the fourth wave of data collection, these questions changed from how often in the past month participants had engaged in an activity to how often in the past year. The question on the frequency of participating in a religious organization in wave 4 and wave 5 include an additional answer option of “Almost every month”, along with four options that matched the previously used frequencies except for the option “Less often” now referring to “Less often than monthly”. To maintain a scale equal to the absolute frequency asked in the previous waves, we coded the responses “Almost daily” and “Almost every week” as 1, and made our reference category “No participation or less than weekly”. Second, praying was assessed by the question “Thinking about the present, how often do you pray?” and captures the personal aspect of religious involvement. We categorized responses into “Never”, “Less often than weekly”, “Weekly or a couple of times a week”, “Daily” and “More than once a day”. Third, religious education was assessed only when entering the survey by the question “Have you been educated religiously by your parents?”. We include the measure of religious education as a proxy for “restful” religiousness when looking at religiousness and its relationship with depressive symptoms.

#### Socio-demographic and health controls

Gender, country and educational level are included as time constant covariates. Highest educational level is grouped according to the International Standard Classification of Education [44] into primary school (classifications 0–1); lower secondary (classification 2); higher secondary (classifications 3–4); and tertiary education (classifications 5–6). The set of time-varying covariates which are all repeatedly measured across all four waves includes age, marital status, economic strain, four measures of physical health and info on personal depression history. Age is categorized in three age groups: 65–74, 75–79, 80–84 and 85 years old or older. Marital status is grouped in married, divorced/separated/never married, and widowed. Economic strain is a subjective indicator of financial distress (“Thinking of your household's total monthly income, would you say that your household is able to make ends meet?”), dichotomized into ‘with difficulties’, and ‘easily’. The first indicator of physical health status is a binary variable indicating whether an individual reports at least one limitation in activities of daily living (ADL). These activities comprise tasks that are necessary for personal care, like showering and self-feeding. Second, a variable indicating whether a respondent reports at least one limitation in instrumental activities of daily living (IADL). These are tasks essential to maintain a living environment, such as shopping for groceries and preparing a hot meal. Third, self-rated health was assessed by a generic question dichotomized into good versus poor. Fourth, long-term health problems are assessed by respondents indicating whether they have any long-term health problems, illness, disability or infirmity. Finally, personal history of depression is assessed by questions on whether the respondent ever suffered from a major depression.

### Statistical methods

In OLS regression it is assumed that the observations in the sample are independent of each other. However, the same respondents contribute two or more records to our dataset which makes a direct application of OLS to our data inappropriate. To solve this dependence problem, we analyze the changes in depressive symptoms over time and explore the effects of time-varying measures of religiousness on individual depression outcomes by means of random and fixed-effects models. This way, the total number of waves is allowed to vary among respondents and respondents who are not surveyed in all waves are retained in the analyses. First, we estimate random effects models to estimate the effect of religiosity on depressive symptoms. Random effects models assume that unobserved differences between individuals that are constant over time are random [45], and allow us to assess the effects of both time-varying and time-constant variables.

The second specification is based on individual fixed-effects regressions which use within-individual changes in religiousness while controlling for all measured and unmeasured time-invariant confounders that differ across individuals [46,47]. An appealing feature of a fixed effects model is that the coefficient estimates are not biased by measured or unmeasured time-invariant confounders, such as genetic differences and personality traits, which may play an essential role in the relation between religiosity and mental health. Failure to control for such characteristics may yield biased estimations of the observed association. Our fixed effects models can be expressed as

$$y_{it}=\beta_{0}+\beta_{1}x_{it}+\beta_{2}z_{it}+\alpha_{i}+\varepsilon_{it}$$ (1)

where *y*_*it*_ indicates the score on the depression scale for individual *i* at wave *t*. A set of measurements of religiousness (i.e. participating in a religious organization and frequency of praying) which varies over time is represented by the vector *x*_*it*_ and a set of time-varying control variables *z*_*it*_ with each set their respective coefficients *β*_*1*_ and *β*_*2*_. Coefficient *α*_*i*_ is treated as a constant effect, which controls for time-invariant individual characteristics, and *ε*_*it*_ is the within-individual error term. The key distinction between the random and fixed effects model is whether *α*_*i*_ is assumed to be correlated with the other covariates in the model. In a random effects model, *α*_*i*_ is assumed to be independent of the other covariates, while in a fixed effects model *α*_*i*_ represents the unmeasured confounding [47].

We follow a stepwise approach to build the models, starting with models that control for age and wave only. We then incorporate time-varying, which includes marital status, financial situation, ADL, IADL, self-rated health, suffering from a long-term health problem, history of depression, and time-constant controls, which includes gender, educational level, being religiously educated and country. We estimate individual clustered robust standard errors for all estimates. All analyses are conducted using Stata version 15.1 (StataCorp LG, College Station, Texas). The code supporting this article is available upon request from the authors.

## Results

Sample baseline characteristics are summarized in Table 1. The mean number of depressive symptoms is 2.45 (SD = 2.31) with 26.7% of the respondents reporting a score ≥ 4, indicating a clinical depression. The vast majority of respondents (88.8%) does not participate in a religious organization or does so less than monthly. In terms of praying, 27.4% never prays, 15.4% prays less often than weekly, 19.2% weekly or a couple of times a week, 21.9% daily and 16.1% prays more than once a day. The mean age is 71 (SD = 6.35). The majority of our respondents are Catholic (43.9%) or Protestant (26.4%). Only 0.8% is Muslim, and 6.7% is Jewish. 10.3% stated that they do not belong or feel attached to a religion. Approximately 46.9% of the respondents are men, 56.5% suffers from a long-term health problem, and 25.3% is widowed.

**Table 1:** Baseline characteristics of selected respondents aged 65 years or older in the survey of health, ageing and retirement, 2004–2013.

Characteristic		No. of respondents (n = 7,719)^b^	%
Health indicators			
	EURO-D score^a^	2.45 (0.03)	
	EURO-D score ≥ 4	2060	26.7
	Having limitations with ADL^a^	1052	13.6
	Having limitations with IADL^a^	1828	23.7
	Self-reported bad or poor health	5884	76.5
	Long-term health problems	4358	56.5
Religiosity indicators			
	Religiously educated	6036	78.2
	Never or less than weekly taken part in a religious organization (church, synagogue, mosque, etc.).	6855	88.8
	Almost daily or weekly taken part in a religious organization (church, synagogue, mosque, etc.).	864	11.2
	Prays never	2117	27.4
	Prays less often than weekly	1189	15.4
	Prays weekly or a couple of times a week	1481	19.2
	Prays daily	1688	21.9
	Prays more than once a day	1244	16.1
	Protestant	2011	26.2
	Catholic	3362	43.9
	Orthodox	802	10.5
	Jewish	515	6.7
	Muslim	59	0.8
	Other	124	1.6
	None	790	10.3
Demographic characteristics			
	Age, years^a^	73.25 (0.07)	
	Female	4172	54.1
	Male	3547	46.9
	Married, registered partnership and living together	5056	65.5
	Separated, divorced or never married	712	9.2
	Widowed	1951	25.3
Socioeconomic characteristics			
	Primary school (ISCED 0–1)	3272	42.7
	Lower secondary (ISCED 2)	1327	17.3
	Higher secondary (ISCED 3–4)	2000	26.1
	Tertiary education (ISCED 5–6)	1058	13.8
	Having difficulties to make ends meet	2120	38.2

Abbreviations: ADL, activities of daily living; EURO–D, depression symptoms scale running from 0 to 12; IADL, instrumental activities of daily living; ISCED, International Standard Classification of Education.

^a^ Expressed as mean values (standard deviations).

^b^ Sample sizes vary due to missing values for ‘Having difficulties to make ends meet’ (n = 5,547), educational level (n = 7,656), and self-rated health (n = 7,697).

It is important to note that both the mean score on the EURO-D scale and how people practice their religiousness varies across the 11 countries examined (see Table 2). Looking at the depression score, Switzerland and Denmark stand out with the lowest mean of respectively 1.78 (SD = 0.10) and 1.79 (SD = 0.09). The highest score is found in Spain with a mean of 3.71 (SD = 0.10), followed up by Israel with a mean of 2.99 (SD = 0.10). Turning to public religious participation, we find the highest proportion of people participating almost daily or every week in a religious organization in Greece with 35.44% (SD = 0.02) and Israel with 16.51% (SD = 0.01), and the lowest ones in Italy with 3.66% (SD = 0.01) and Denmark with 4.02% (SD = 0.01). On the contrary, when looking at private religious participation, we see that Italy has one of the highest proportion of respondents indicating that they pray at least weekly with a percentage of 74.87 (SD = 0.02). Only Greece scored higher with 89.65% (SD = 0.01). People pray the least in Sweden and Denmark, with respectively 28.10% (SD = 01) and 40.4% (SD = 0.02) of the respondents praying at least on a weekly basis.

**Table 2:** Baseline mean depression scores and public and private religious participation by country.

	EURO-D				Public religious participation^a^				Private religious participation^b^			
	Mean	95% CI			%	95% CI			%	95% CI		
Austria	2.23	2.046	,	2.406	14.24	0.115	,	0.170	65.47	0.616	,	0.691
Germany	2.16	2.015	,	2.314	7.72	0.059	,	0.096	48.86	0.454	,	0.523
Sweden	2.08	1.958	,	2.211	6.81	0.052	,	0.084	28.10	0.253	,	0.311
Netherlands	2.10	1.956	,	2.244	8.41	0.064	,	0.104	56.63	0.531	,	0.601
Spain	3.71	3.511	,	3.910	8.64	0.066	,	0.106	67.96	0.645	,	0.711
Italy	2.84	2.647	,	3.033	3.66	0.022	,	0.052	74.87	0.713	,	0.782
Denmark	1.79	1.615	,	1.956	4.02	0.022	,	0.058	40.40	0.360	,	0.450
Greece	2.65	2.483	,	2.816	35.44	0.321	,	0.388	89.65	0.873	,	0.916
Switzerland	1.78	1.586	,	1.977	7.74	0.047	,	0.108	65.32	0.597	,	0.705
Belgium	2.26	2.137	,	2.379	6.72	0.052	,	0.082	58.22	0.553	,	0.611
Israel	2.99	2.785	,	3.196	16.51	0.136	,	0.194	38.01	0.343	,	0.418

^a^ Participating at least weekly in a religious organization (church, synagogue, mosque, etc.).

^b^ Praying at least weekly

The estimates of model 1 (Table 3) show that participation in religious organizations (β = -0.219, 95% CI: -0.344, -0.094) is significantly associated with fewer depressive symptoms, after adjusting for age and survey wave. Praying daily (β = 0.428, 95% CI: 0.303, 0.554) and more than once a day (β = 0.588, 95% CI: 0.445, 0.731) are associated with a higher score on the depression scale. Results of model 2 show that being a woman (β = 0.463, 95% CI: 0.362, 0.564), widowed (β = 0.285, 95% CI: 0.165, 0.404), having financial difficulties (β = 0.488, 95% CI: 0.387, 0.588), limitations in activities of daily living (β = 0.811, 95% CI: 0.657, 0.964), limitations in instrumental activities of daily living (β = 0.895, 95% CI: 0.774, 1.015), long-term health problems (β = 0.399, 95% CI: 0.313, 0.484), poor self-reported health (β = 0.479, 95% CI: 0.391, 0.567), and having suffered from depression in the past (β = 1.201, 95% CI: 1.083, 1.320) are all associated with higher levels of depressive symptoms. Focusing on the measures of religiosity, we see that public religious involvement is not significantly associated anymore with fewer depressive symptoms (β = -0.092, 95% CI: -0.223, 0.039). The private aspect of religiosity, praying more than once a day (β = 0.150, 95% CI: 0.003, 0.298) is significantly associated with more depressive symptoms, after controlling for several socio-demographic and health controls, history of depression, country, and survey wave.

**Table 3:** Random effects models, religiousness and depressive symptoms among respondents aged 65 years or older, survey of health, ageing and retirement, 2004–2013.

Characteristic			Model 1^a^				Model 2^a^			
			β	95% CI			β	95% CI		
Exposure of interest										
	Participating in religious organization^b^		-0.219	-0.344	,	-0.093	-0.092	-0.223	,	0.039
	Frequency of praying^c^									
		Prays less often than weekly	0.017	-0.103	,	0.138	-0.093	-0.223	,	0.037
		Prays weekly or a couple of times a week	0.068	-0.052	,	0.189	-0.125	-0.255	,	0.005
		Prays daily	0.428	0.303	,	0.554	0.066	-0.065	,	0.298
		Prays more than once a day	0.588	0.445	,	0.73	0.150	0.003	,	0.298
	Religiously educated		-0.013	-0.043	,	0.017	0.024	-0.007	,	0.055
Socio-demographic and health controls										
	Age^d^									
		70–74 years	0.092	-0.002	,	0.186	-0.028	-0.131	,	0.074
		75–79 years	0.377	0.262	,	0.492	0.189	0.069	,	0.309
		80–84 years	0.791	0.651	,	0.930	0.266	0.122	,	0.410
		85 years and older	0.989	0.798	,	1.180	0.160	-0.039	,	0.359
	Educational level^e^									
		Lower secondary (ISCED 2)					-0.101	-0.239	,	0.037
		Higher secondary (ISCED 3–4)					-0.123	-0.250	,	0.003
		Tertiary education (ISCED 5–6)					-0.236	-0.378	,	-0.094
	Female						0.463	0.362	,	0.564
	Divorced, separated or never married^f^						0.111	-0.036	,	0.257
	Widowed^f^						0.285	0.165	,	0.404
	Having difficulties to make ends meet^g^						0.488	0.387	,	0.588
	ADL						0.811	0.657	,	0.964
	IADL						0.895	0.774	,	1.015
	Self-rated health^h^						0.479	0.391	,	0.567
	Long-term health problem						0.399	0.313	,	0.484
	History of depression						1.201	1.083	,	1.320

Abbreviations: ADL, activities of daily living; CI, confidence interval; IADL, instrumental activities of daily living; ISCED, International Standard Classification of Education.

^a^ Models included survey-wave and country effects.

^b^ Reference category: Never participates or less than monthly.

^c^ Reference category: Prays never.

^d^ Reference category: 65–69 years.

^e^ Reference category: Primary school ISCED (0–1).

^f^ Reference category: Married, registered partnership and living together.

^g^ Reference category: Easily and fairly easy to make ends meet.

^h^ Reference category: excellent and good self-rated health.

Results from the fixed-effects models are presented in Table 4. Here, we control for unobserved individual heterogeneity that may be correlated with our explanatory variables. Model 3 only controls for age and survey wave. The results show that changes in public (β = 0.097, 95% CI: -0.082, 0.275) and private (β = 0.124, 95% CI: -0.122, 0.370) religious involvement are not significantly associated with changes in depressive symptoms. In model 4, we see that public religiousness (β = 0.275, 95% CI: 0.075, 0.475) is significantly related to more depressive symptoms. No significant effects are found for private religious involvement (β = -0.173, 95% CI: -0.471, 0.124). Furthermore, changes in financial difficulties (β = 0.239, 95% CI: 0.068, 0.410), activity limitations in daily living (β = 0.452, 95% CI: 0.201, 0.703), limitations in instrumental activities of daily living (β = 0.692, 95% CI: 0.509, 0.874), and self-rated health (β = 0.157, 95% CI: 0.019, 0.295), and suffered from a depression in the past (β = 0.622, 95% CI: 0.443, 0.801) are associated with increases in depressive symptoms.

**Table 4:** Fixed-effects models, religiousness and depressive symptoms among respondents aged 65 years or older, survey of health, ageing and retirement, 2004–2013.

Characteristic			Model 3^a^				Model 4^a^			
			β	95% CI			β	95% CI		
Exposure of interest										
	Participating in religious organization^b^		0.097	-0.082	,	0.275	0.275	0.075	,	0.475
	Frequency of praying^c^									
		Prays less often than weekly	-0.040	-0.213	,	0.134	-0.139	-0.354	,	0.076
		Prays weekly or a couple of times a week	-0.054	-0.251	,	0.144	-0.181	-0.426	,	0.062
		Prays daily	0.086	-0.139	,	0.311	-0.145	-0.416	,	0.126
		Prays more than once a day	0.124	-0.122	,	0.371	-0.173	-0.471	,	0.124
Socio-demographic and health controls										
	Age^d^									
		70–74 years	-0.155	-0.309	,	-0.001	-0.129	-0.321	,	0.062
		75–79 years	-0.231	-0.481	,	0.019	-0.115	-0.417	,	0.186
		80–84 years	-0.050	-0.414	,	0.314	-0.130	-0.561	,	0.300
		85 years and older	-0.021	-0.535	,	0.493	-0.050	-0.669	,	0.570
	Divorced, separated or never married^e^						0.054	-3.207	,	1.849
	Widowed^e^						0.489	-0.049	,	1.030
	Having difficulties to make ends meet^f^						0.239	0.068	,	0.410
	ADL						0.452	0.201	,	0.703
	IADL						0.692	0.509	,	0.874
	Self-rated health^g^						0.157	0.019	,	0.295
	Long-term health problem						0.098	-0.039	,	0237
	History of depression						0.622	0.443	,	0.801

Abbreviations: ADL, activities of daily living; CI, confidence interval; IADL, instrumental activities of daily living.

^a^ Models included survey-wave fixed-effects.

^b^ Reference category: Never participates or less than monthly.

^c^ Reference category: Prays never.

^d^ Reference category: 65–69 years.

^e^ Reference category: Married, registered partnership and living together.

^f^ Reference category: Easily and fairly easy to make ends meet.

^g^ Reference category: excellent and good self-rated health.

## Discussion

This study adds longitudinal evidence of religiousness and its effects on depression using data from 11 European countries. Our findings confirm that religious behavior is associated with depressive symptoms in old age and that the strength and direction of this association varies among different aspects of religiosity. The results of our random effects models show that participation in religious organizations (public religious involvement) is associated with fewer depressive symptoms, while higher frequency of praying (private religious practice) is associated with higher levels of depressive symptoms. However, the protective effect of public religious involvement vanishes when we take due account of possible confounders (financial difficulties, sex, ill health, disability, education, country and personal history of depression), suggesting a spurious relationship. For example, respondents who less frequently attend a church service report higher levels of depressive symptoms merely due to health problems, or because they suffered from a depression in the past. Furthermore, we find that sex, educational level, perceived financial situation and physical health all significantly affect depressive symptoms.

The findings complement the current US-centered literature on religiousness and mental health by looking at the European context, and its cross-national variations in mean scores on the EURO-D scale and religious participation. Looking at depression scores, we see that Southern-Europe reports higher levels of mean EURO-D scores, compared to Northern and Western-Europe, confirming findings from earlier studies [43,48]. Additionally, there are also regional differences in how people practice their religion. Highest levels of public participation are found in Greece and Israel. Although Italy reported the lowest levels of service attendance, they have one of the highest levels of private religious participation compared to the rest of Europe. Only Greece has a higher proportion of people praying at least weekly, and can be seen as ‘*the most religious’* among the 11 countries examined. These results are in line with previous findings for the Mediterranean countries (i.e. Greece, Spain and Italy) which exhibit the highest levels of private religious activity compared to elsewhere in Europe [37].

A key challenge in this field of research is selection: is the association between religiousness and mental health the result of religiousness influencing mental health, or are there unobserved characteristics that may confound their relationship? We attempt to address this issue by means of fixed-effects modelling which accounts for all time-constant characteristics that may influence the association between religiosity and depressive symptoms, without explicitly measuring them. This ensures that our estimates are not biased by time-invariant confounders such as genetic differences and personality traits, which may play an essential role in the relation between religiosity and mental health. Focusing on within-person variation, we find that changes in public religious behavior predict changes in depressive symptoms in a positive way, with higher levels of church attendance predicting higher levels of depressive symptoms. This finding seems counter-intuitive with previous longitudinal research on religiousness and depression in the U.S. [28, 30–32]. It might be that in Europe, public religious participation is losing its significance, due to increasing religious individualization [39], which might explain our contradictory findings. In that case, merely participating in these activities may not be sufficient to benefit from the social support offered by religious organizations. It may be necessary to be a “true believer” [49] or to be “more religious” [8] and internalize religious meanings and values also privately, and not solely by attending religious services. Yet, no significant effect is found for praying when looking at within-persons variations in praying behavior, which is in contrast with the results of our random effects model but in line with previous research suggesting much weaker or no associations between private practice measures of religiosity and health [33]. The different estimates of the private aspect of religiosity between the random effects and fixed-effects approaches suggest a selection effect i.e. individuals with higher scores on the depression scale are more likely to pray multiple times a day, and continue to do so, compared with less depressed respondents. It is plausible that prolonged reliance on prayer may lead to neglecting professional help, which subsequently leads to rising levels of depressive symptoms. However, we have no evidence that changes in praying behavior are accompanied by changes in the number of depressive symptoms.

Our study has several advantages over the current state of the literature. First, we use a large, representative, longitudinal sample of older Europeans, as many of the studies on religiousness and old age depression are based on unrepresentative sample, which for example, include only older adults treated in a mental health [20, 21] or clinical setting [50], or focus on the U.S. [28] like the majority of the research on depression and religiousness [33]. Second, we have repeated measures on participation in religious organizations and frequency of praying, in addition to information on whether the respondent was religiously educated, which we considered simultaneously, as these different aspects of religiosity are found to affect depression in different ways [8, 10, 38]. Third, most evidence for the relationship between religiosity and depression is based on cross-sectional designs, precluding causal inference [9]. Using fixed and random effects models, we are able to study specifically the effect of changes in religiosity on changes in depressive symptoms. To date, the issue of which estimator is preferred for survey panel models has not been fully resolved yet. The choice of model is usually assessed by means of the Hausman test, which tests whether the individual-specific fixed-effects modelled in random effects are correlated with the exposure variables [51]. We rejected the null hypothesis that they are not, which makes a fixed-effects model more appropriate. However, the outcome of the Hausman test is not the only aspect to take into account when choosing the appropriate model. In the context of mental health and depression, we cannot be sure that the model we are testing includes all variables that are likely to jointly influence depression and religious involvement. It is plausible that some omitted sources of variation in depressive symptoms correlate with our predictors which makes a fixed-effects model preferable as well. Variables like genetics or personal characteristics, are difficult to measure and can, when not included in the model, produce a specification error in a random effects model [45,46]. We see the use of both methods as a contribution to the research on depressive symptoms and religiousness at old age, as with the random effects models we are able to estimate the effects of time constant characteristics, while the fixed effects models accounts for all unmeasured time constant confounding.

Some limitations should be acknowledged. First, while we do control for unmeasured time-constant confounding in our fixed effects models, unmeasured time-varying confounding remains a potential source of bias. Therefore the estimates may not be interpreted as causal. Changes in religiousness may be correlated with changes in other variables associated with depressive symptoms. For example, older persons may increase their religious involvement after the occurrence of certain life-events, such as the death of a relative, or the birth of a grandchild. Second, in a fixed effects model, the current values of the error term are prohibited to be correlated with past, present and future values of the independent variables [47]. However if current scores on the depression scale affect future religious behavior, the error term is necessarily correlated with future religiousness. This assumption of strict exogeneity can be problematic as individuals suffering from a major depressive disorder are found to be more likely to subsequently stop attending religious services [26,28]. Although we addressed this by controlling for history of depression, the possibility of reverse causation cannot be fully ruled out in a fixed effect setting. Third, a fixed-effects model is based only on the fraction of respondents who change their exposure during the follow-up period. This could potentially lead to large standard errors, when there is little variation in the predictor variables. In our sample, we see that between wave 1 and wave 2, 10.94% of the respondents who participated in both waves changed their participation in religious activities. Between wave 2 and wave 4, this percentage increased to 11.29%. Between wave 4 and 5, 13.79% of the respondents who participated in both waves reported a change on our measure of religious participation.

This study extends prior rigorous research on the relationship between depressive symptoms and religious involvement in old age by assessing their longitudinal relationship in a representative sample of older Europeans. Our analyses demonstrate that public and individual religious involvement are associated with depressive symptoms, but in different ways. Public religious involvement measured as participation in religious activities is associated with fewer depressive symptoms, but this relationship appears to be spurious. When focusing on within-persons changes, we find that changes in church attendance are associated with higher levels of depressive symptoms, even after controlling for changes in health and financial status, history of depression and all time-constant characteristics. Regarding private religious involvement, praying is associated with more depressive symptoms. Yet, our findings suggest that this is due to a selection effect. Our findings do not provide evidence that public policy and healthcare interventions to support religious behavior, both public or private, are effective in reducing depressive symptoms in old age.
